# Food preference and foraging activity of ants: Recommendations for field applications of low-toxicity baits

**DOI:** 10.1093/jis/14.1.48

**Published:** 2014-01-01

**Authors:** Casper Nyamukondiwa, Pia Addison

**Affiliations:** 1 Department of Conservation Ecology and Entomology, Faculty of AgriSciences, Stellenbosch University, Private Bag X1, Matieland 7602, South Africa; 2 Present address: Department of Earth and Environmental Sciences, Faculty of Science, Botswana International University of Science and Technology (BIUST). Private Bag BO 041 Bontleng, Gaborone, Botswana

**Keywords:** bait formulation, bait station densities, integrated pest management, *Planococcus ficus*

## Abstract

Control of ants using baits of low toxicity cannot be effective without knowledge of bait distribution patterns and bait station densities, which are determined by ants’ foraging activities. Furthermore, the success of toxic baits also depends upon attractiveness of bait carriers. Here, we assessed ground and vine foraging activity and food preferences for the three ant species ( *Linepithema humile* (Mayr) (Hymenoptera: Formicidae), *Anoplolepis custodiens* (F. Smith) and *Crematogaster peringueyi* Emery) under field conditions. We found that *L. humile* ’s vineyard foraging activity was high and that movement of ant bait by *C. peringueyi* and *A. custodiens* in the vineyard was relatively low. Consequently, more bait stations need to be dispensed for more effective control of *C. peringueyi* and *A. custodiens* than for *L. humile* . Different bait densities are discussed for the various ant species. Food preference trials indicated that vineyard foraging ants preferred wet bait attractants over dry ones, making liquids the most ideal carriers for baiting these ants. *Linepithema humile* was attracted to 25% sugar water, while *C. peringueyi* was attracted to both 25% sugar water and honey. *Anoplolepis custodiens* was attracted to tuna but was also attracted to 25% sugar water. Thus, future bait formulations should be tailor made to suit these specific food requirements if baits are to be successful in ant pest management.

## Introduction

In ineyards, the mutualism between ants and the vine mealybug, *Planococcus ficus* (Signo-ret) (Hemiptera: Pseudococcidae), results in population explosions of both insects, thereby causing pest injury and economic losses ( Kriegler and Whitehead 1962 ; Way 1963 ; Myburgh et al. 1973 ). The Argentine ant, *Linepithema humile* (Mayr) (Hymenoptera: Formicidae), the cocktail ant, *Crematogaster peringueyi* Emery, and the common pugnacious ant, *Anoplolepis custodiens* (F. Smith), are the major pest ants associated with the vine mealybug in South African vineyards ( Addison and Samways 2000 ). *Linepithema humile* is a cosmopolitan, invasive pest in agricultural and urban areas ( Suarez et al. 2001 ) that nests predominantly in the soil, while *C. peringueyi* and *A. custodiens* are of southern African origin, the former nesting in the canopy and the latter in the soil ( Addison and Samways 2000 ). Current recommendations for control include the use of chemical stem barriers, which target only ground nesting species by preventing access into the canopy ( Addison 2002 ). This control method is designed to break the mutualism between the ants and mealybugs so that natural enemies, primarily encyrtid wasps, can effectively control *P. ficus* without ant interference ( Samways 1990 ).

There is no documented control method for vine-nesting species in South Africa, but low-toxicity baits could be a viable option, as the recruitment to food sources and subsequent food-sharing behavior of ants can be used to distribute toxicant through the colony no matter where the colony is located ( Hooper-Bui and Rust 2000 ; Rust et al. 2004 ). Effective control of South African ants using baits of low toxicity is limited by inadequate knowledge on ant foraging activity and optimum bait station density. Optimal density of bait stations per unit area may vary depending on the ant species and the level of ant infestation. If ants have long foraging distances, then bait density could be lower than if ants have short foraging trails. Daane et al. (2006) dispensed liquid baits at 85–620 baits/ha against *L. humile* in Californian vineyards. At high densities, these baits resulted in fewer *L. humile* , fewer mealybugs, and less damage to grape clusters. Ant foraging ranges and behavior are affected by a number of factors, such as temperature, circadian rhythm, competition, food availability, food particle size, and photoperiod ( Oster and Wilson 1978 ; Hölldobler and Wilson 1990 ). Markin (1968) , for example, found that *L. humile* workers foraged up to 45 m away from the nest. Similarly, Ripa et al. (1999) found up to 21% of marked *L. humile* 54 m from the feeding station. Vega and Rust (2001 , 2003) found marked ants up to 61 m from feeding stations. However, ant foraging activity in urban settings may differ from a vineyard because of varying abundance of food resources. Level of infestation by honeydew-producing mealybugs would presumably also impact foraging activity. Although *L. humile* can forage for long distances, these ants generally nest in close proximity to food sources and relocate nests when nearby food sources become exhausted ( Suarez et al. 2001 ; Tsutsui et al. 2003 ; Heller and Gordon 2006 ). In South African citrus orchards, *A. custodiens* has been observed to forage 111 m away from their nest during peak honeydew production periods and up to 50 m in April, when the mealybugs are abundant ( Steyn 1954 ). No research has been carried out to ascertain *C. peringueyi* foraging activity.

Different ant species have different dietary requirements, and consequently this must be considered during ant bait formulation. Effective proportions of fat, carbohydrates, and proteins in the bait may differ between species and with the colonies’ nutritional needs ( Rust et al. 2000 ). Physical state of the bait, liquid versus solid, and particle size also affect the rate of collection of the bait ( Hooper-bui and Rust 2000 ). Most of the research on ant food preference has been done on *L. humile* ( Baker et al. 1985 ). To date, no food preference studies have been done in South African vineyards to determine ideal food requirements for *L. humile* , *A. custodiens* , or *C. peringueyi* .

When dissolved in 25% sugar water, preliminary investigations showed that low toxicity baits were non-repellent to foragers ( Nyamukondiwa and Addison 2011 ), palatable, and exhibited sufficient delayed toxicity to allow trophallaxis ( Stringer et al. 1964 ). The present research was aimed at determining (i) the foraging activity (that is the distance each ant travels from its nest to the source of food) of vineyard ants ( *L. humile* , *C. peringueyi* , and *A. custodiens* ) using liquid baits labeled with a marker and (ii) whether specific bait matrices (different food attractants) had different attractiveness to the three ant species under field conditions. Results of this trial will help us to better determine bait station density, bait distribution patterns, and the best attractant to use when formulating toxicants for the control of these three ant species. This information is critical for the practical use of ant baits in agroecosystems.

## Materials and Methods

### Study sites

Preference trials were carried out during austral spring (October 2007), as this is the optimum time for application of baits because ant populations are low ( Nelson and Daane 2007 ). Foraging activity evaluations were carried out during summer (December 2007, January and February 2008) in the Stellenbosch Winelands region. Ideally this should also have been conducted in spring, but selection of a suitable marker for the ants delayed this experiment. *Linepithema humile* trials were carried out at Joostenberg farm (33.80S, 18.81E), *C. peringueyi* at La Motte farm (33.88S, 19.08E), and *A. custodiens* trials were carried out at Plaisir de Merle farm (33.87S, 18.94E). Drip irrigation was used in all vineyards. The Plaisir de Merle site was a trellised white Chenin blanc vineyard, the La Motte vineyard comprised trellised white Chenin blanc grapes, and the Joostenberg farm had trellised red Pinotage wine grapes. In all vineyards, standard sprays of alpha-cypermethrin and chlorpyrifos were routinely used to control ants during the growing season.

### Experiment 1: Determination of ant foraging activity

**Trial layout and sampling methods.** Five bait stations were placed 10 m apart ( Daane et al. 2006 ) on one side of the perimeter of a vineyard block approximately 100 m x 100 m, and ants were allowed to feed on 25% sugar solution that had been labeled with 0.25% calco red (N-1700 ^®^ , Passaic Color and Chemical Company, Paterson, New Jersey). These foraging activity trials were carried out on three dates (December 2007, January and February 2008). The calco red labeled sugar water was soaked in cotton wool and held in place on Petri dishes. The concentration of calco red dye used followed recommendations from literature (see discussions in Vega and Rust 2001 ). Ants were allowed to forage on the calco red labeled sugar water for one week before ant sampling was done. The labeled sugar was replenished once during the week because the bait easily dried out due to crystallization of the sugar component.

Seven days after placing baits into vineyards, thirty pitfall traps were arranged in five transects of 1, 2, 4, 8, 16, and 32 m from the bait stations running along the vine rows. All pitfalls ran along vine rows where the baits were placed. Each bait station had one transect associated with it, replicated five times (over five rows 10 m apart) to give a total of 30 traps. Pitfall traps consisted of plastic containers (35 mm diameter x 60 mm height). These were drilled into the soil, and the soil surface was leveled so that the trap rim was flush with the soil surface. Approximately 4 mL of three parts concentrated glycerol and seven parts 70% ethyl alcohol were placed in each of the pitfall traps to preserve the captured ants (following Majer 1978 ). This liquid is relatively non-volatile. Pitfall traps were left in the vineyard for 48 hours, after which the traps and their catch were collected for laboratory analysis.

When pitfall traps were being collected, tuna baits were used to sample arboreal ant species, as these would not have been sampled in pitfall traps and are also potential pest species. *Crematogaster peringueyi* forage and nest in the vine canopy, while the other species mentioned nest primarily on the ground. Roughly 5 cm ^3^ of shredded tuna chunks were placed in small plastic containers (70 mm diameter x 7 mm height) at the crutch of vines above each of the pitfall traps along each of the five transects. Ants were left to forage on the tuna for 30 minutes, after which all ants feeding on the tuna bait were collected by sweeping them in different containers containing 70% ethyl alcohol as a preservative.

Ant samples collected from both pitfall and tuna traps were taken to the laboratory for analysis. Calco red positive ants were detected by crushing the ant’s abdomen on white paper towels and observing its coloration through a microscope. A pink coloration of the abdomen indicated the presence of calco red.

### Experiment 2: Food preference assessments

Eight food attractants (bait matrices) were assessed for their attractiveness to *L. humile* , *C. peringueyi* , and *A. custodiens* during spring (October) 2007. These included: (1) 25% sugar solution; (2) agar (in 25% sugar solution) (Warren Chemical Specialities, www.warrenchem.co.za ); (3) tuna (Pick’n pay, www.picknpay.co.za ); (4) honey (Fleures ^®^ Honey Products, Pretoria, South Africa); (5) dog food (Boss ^®^ chicken beef and beef platter, Promeal Private Limited, South Africa); (6) dry fish meal; (7) dry sorghum grit; (8) 25% peanut butter (in distilled water) (Nola Yum Yum ^®^ , Nola, Randfontein, South Africa). Tests were conducted in vineyards using choice test arenas. Choice test arenas were made of round plastic containers (270 mm diameter by 65 mm height) with eight plastic tubes (125 mm long by 8 mm diameter) that extended through eight openings at 45° in the inside of the choice test arena, as described in Tollerup et al. (2004) . The tubes directed all ants to the center of the choice test arena before they could forage on a bait of their choice. Ants were free to move in and out of the choice test arenas during the test period. Five choice test arenas were used (five replicates), and eight bait matrices, approximately 10 mL, were placed in small Petri dishes, which were randomly assigned to positions in the choice test arenas on the perimeter of the test arena. Active ant nests were selected in the vineyards, and each arena was placed close by, with approximately 10 m distance between arenas. Ants were allowed to forage on the baits, and the experiment was replicated at five nests for each of the three ant species. Each experiment started at ~08:00 hours in the morning, after which ant counts occurred hourly for four hours following food deployment. Specifically, this was done by counting the number of ants sitting at each bait matrice at every hour. For *L. humile* , Rust et al. (2000) reported increased foraging activity in the afternoon compared to mornings, but this may be dependent on microhabitat temperatures, which may differ from in different habitats and has not been tested for *L. humile* , *C. peringueyi* , and *A. custodienns* in a South African context.

### Data collection and analysis

The proportion of ants testing positive for calco red for each of the respective distances of the two trapping methods and for each of the three ant species was calculated. For each distance, and for each of the two trapping methods, data were pooled across the five transects. Since the results (proportion of calco red positive ants) were measured under different conditions (months), using standard ANOVA in this case was inappropriate because it fails to model the correlation between the repeated measures. The proportion of ants that carried the dye labeled sugar water at each of the various distances from the bait source for the two trapping methods was therefore calculated for each ant species using repeated measures ANOVA in Statistica 7 (StatSoft, www.statsoft.com ). Tukey-Kramer’s post hoc tests were used to identify statistically heterogeneous means. During the food preference tests, the number of ants foraging at each bait station was recorded at hourly intervals up to four hours. Before analysis, data were checked for normality and equality of variances using the Shapiro-Wilk test and Hartley-Bartlett tests, respectively, and in all cases these assumptions were met. Results were then subjected to ANOVA in Statistica 7, and Tukey-Kramer’s post hoc test was again used to separate means.

## Results

### Experiment 1: Determination of ant foraging activity

**Figure 1.**
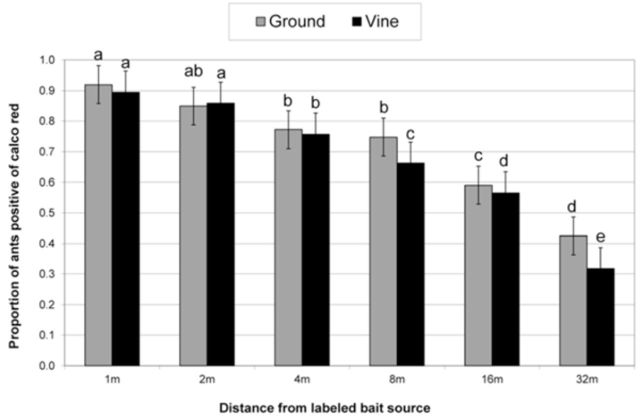
Proportion of *Linepithema humile* positive of a calco red labeled bait source (where 1.0 = 100%) at different distances from the bait source in a vineyard at Joostenberg farm. There was a significant drop in foraging activity after 2 and 8 m from the vine canopy and on the ground respectively. Ground and vine data were analyzed separately, and means with the same letter are not significantly different (Bars represent ±95% CI). High quality figures are available online

***Linepithema humile* .** There were no significant monthly differences in *L. humile* foraging activity during the three different trial dates both on the ground and on the vine ( Table 1 ). However, foraging activity was highly significant both on the ground and in the vine ( Table 1 ), with distance generally decreasing from the bait source for both ground and vine sampled ants, which was to be expected ( Figure 1 ). There was a significant drop in foraging activity after 2 m in the vine canopy and after 8 m on the ground ( Figure 1 ).

**Table 1.**
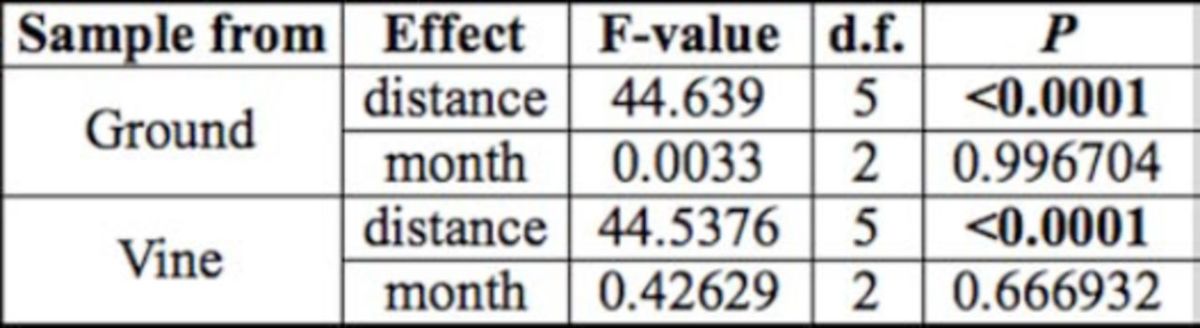
Summary of the effects of distance and month on *Linepithema humile* foraging distance in small field trials. Bold treatments are statistically significant.

**Figure 2.**
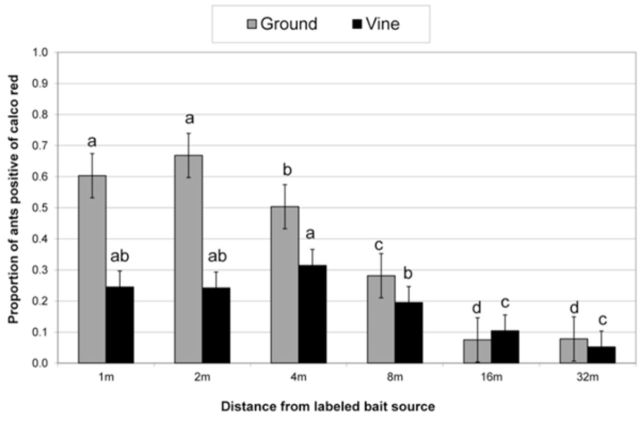
Proportion of *Anoplolepis custodiens* positive of a calco red labeled bait source (where 1.0 = 100%) at different distances from the bait source in a vineyard at Plaisir de Merle farm. Ground and vine data were analyzed separately, and means with the same letter are not significantly different (Bars represent ±95% CI). High quality figures are available online.

***Anoplolepis custodiens* .** The proportion of ants occurring on the ground was generally higher than those occurring in the vine canopy ( Figure 2 ). Nevertheless, a considerable number of ants were caught on the vines, where they were observed tending mealybugs. There were no significant monthly differences in foraging activity during the three trial days (during December, January, and February) both on the ground and on the vine ( Table 2 ). However, foraging distance during the three different months was significant both on the ground and on the vine ( Table 2 ), with distance from the bait source being significant on the ground while on the vine there was no difference in number of marked ants until after 8 m ( Figure 2 ). A significant drop in *A. custodiens* foraging activity occurred on the ground after 2 m ( Figure 2 ).

**Table 2.**
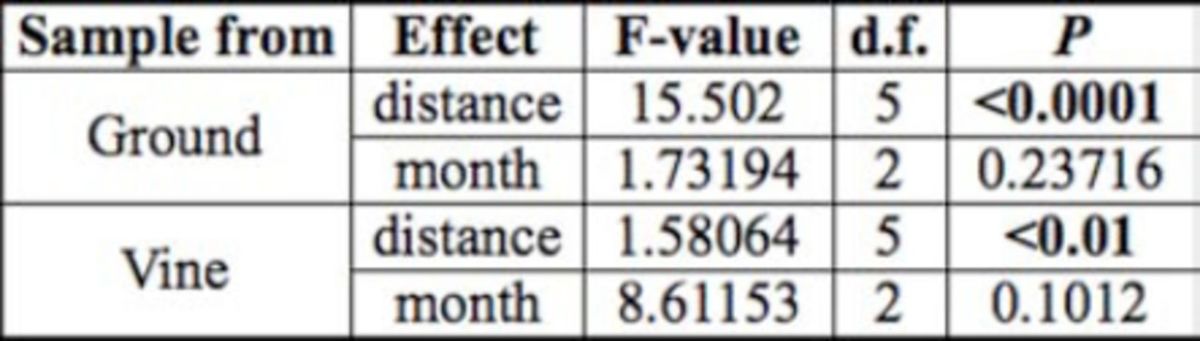
Summary of the effects of distance and month on *noplolepis custodiens* foraging distance in small field trials. Bold treatments are statistically significant.

**Figure 3.**
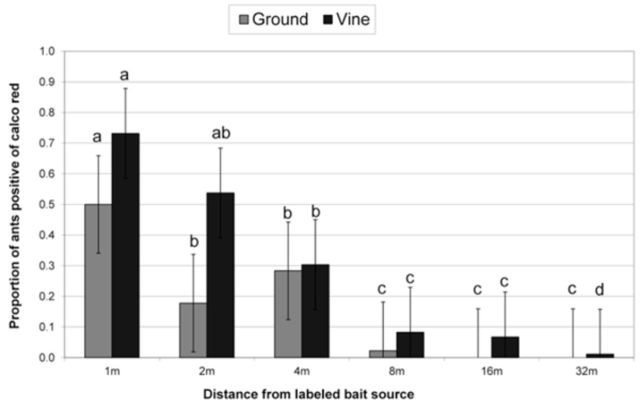
Proportion of *Crematogaster peringueyi* positive of a calco red labeled bait source (where 1.0 = 100%) at different distances from the bait source in a vineyard at La Motte farm. Ground and vine data were analyzed separately, and means with the same letter are not significantly different (Bars represent ±95% CI). High quality figures are available online.

***Crematogaster peringueyi*** Detection of the calco red labeled sugar water again generally decreased with distance from the bait source ( Figure 3 ). The distance foraged by *C. peringueyi* was highly significant for both ground-sampled and vine-sampled ants ( Table 3 ). *Crematogaster peringueyi* were more predominant on the vines as opposed to the ground. There were no monthly differences in *C. peringueyi* foraging activity during the three trial days (during December, January, and February) both on the ground and on the vine ( Table 3 ). Transport of the calco red labeled sugar water dropped significantly after 4 m in the vines and after only 1 m on the ground ( Figure 3 ).

**Table 3.**
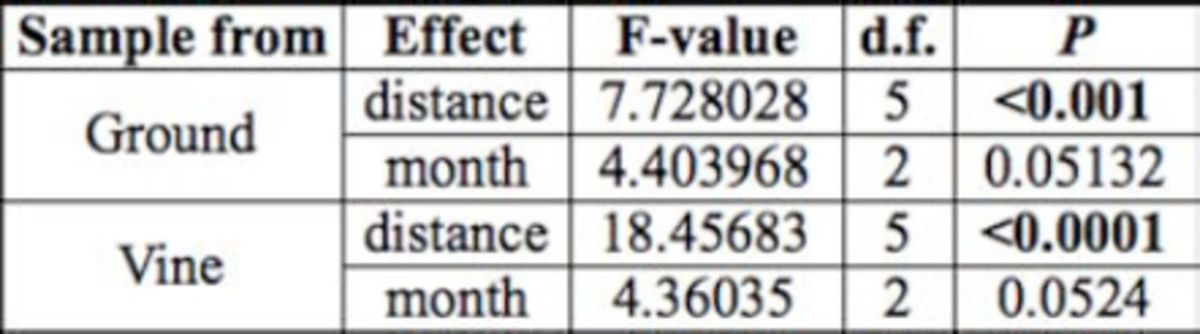
Summary of the effects of distance and month on *Crematogaster peringueyi* foraging distance in small field trials. Bold treatments are statistically significant.

### Experiment 2: Food preference assessments

**Figure 4.**
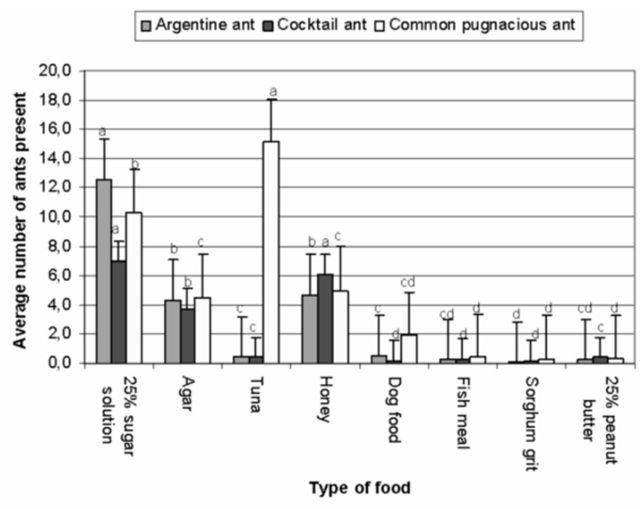
Average number of *Linepithema humile, Crematogaster peringueyi* , and *Anoplolepis custodiens* on different food baits during October 2007. Ant species were analyzed separately, and means with the same letter are not significantly different (bars represent ±95% CI). High quality figures are available online.

**Figure 5.**
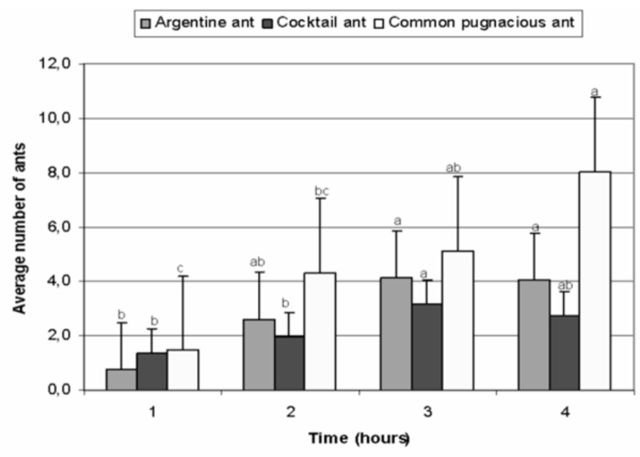
Average number *Linepithema humile, Crematogaster peringueyi* , and *Anoplolepis custodiens* on food baits over time in spring. Ant species were analyzed separately, and means with the same letter are not significantly different (bars represent ±95% CI). High quality figures are available online.

****Linepithema humile**** . Field trials revealed that treatment (baits) and time after bait deployment (measured in hours) were highly significant for this ant species ( Table 4 ). Generally, *L. humile* was more attracted to sugar-based baits as opposed to protein-based baits. A 25% sugar solution was the most attractive bait and significantly differed from the rest of the treatments ( Figure 4 ). *Linepithema humile* significantly preferred liquid baits (25% sugar water, honey, and agar) over solid baits ( Figure 4 ). The number of foraging *L. humile* sig-significantly increased with time ( Figure 5 ).

**Table 4.**
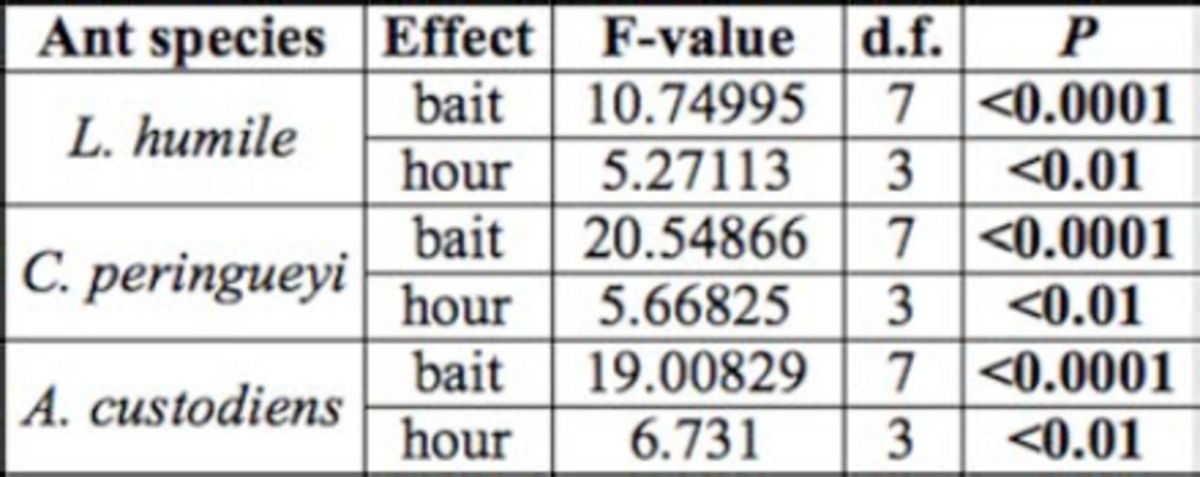
Summary of the effects of bait and hour on food preference for *Linepithema humile* , *Crematogaster peringueyi* , and *Anoplolepis custodiens* . Bold treatments are statistically significant.

***Crematogaster peringueyi* .** There were no significant differences in this ant’s preference for 25% sugar solution as compared to 25% honey. These two baits were the most attractive to *C. peringueyi* and significantly differed from the rest of the treatments ( Figure 4 ). Both bait treatments and time had significant effects on this ant species ( Table 4 ). The number of foraging *C. peringueyi* significantly increased with time ( Figure 5 ).

***Anoplolepis custodiens*** Bait treatments had a significant effect during the field trials ( Table 4 ). *Anoplolepis custodiens* was significantly attracted to tuna more than the other treatments ( Figure 4 ). The number of foraging *A. custodiens* significantly increased over time ( Figure 5 ).

## Discussion

*Linepithema humile* had the highest proportion of ants foraging at 32 m, with *C. peringueyi* having the lowest proportion ( Figures 1–3 ). The former are successful and aggressive competitors that generally dominate rapidly over other ant species ( Hölldobler and Wilson 1990 ). They are unicolonial, meaning that ants from one colony are not aggressive to non-nestmates ( Passera and Keller 1994 ). Markin (1968) found that, after five days, the exchange of worker ants between neighboring nests exceeds 50%. In this manner, *L. humile* can easily form supercolonies that can saturate an entire habitat. Nelson and Daane (2007) also found that *L. humile* is extremely vagile in Californian vineyards, whereas in citrus orchards Markin (1967 , 1968) and Ripa et al. (1999) found that *L. humile* foraging seldom exceeds 61 m from a nest. Our research was limited to 32 m, as placing baits further apart would probably not result in effective control (see Daane et al. 2006 ). *Linepithema humile* foraged equally effectively on the ground and in the vine canopy, as indicated by the proportion of workers caught in each of the traps (pitfalls vs. food baits in the canopy).

The proportion of the arboreal *C. peringueyi* sampled on the ground were very low and inconsistent, confirming their arboreal foraging habits. The majority of ants foraged no further than 4 m from the bait source, both on the ground and in the vine canopy, indicating a very limited foraging area. *Anoplolepis custodiens* indicated a foraging distance of up to 8 m, that distance being where the highest proportion of workers were trapped, with more foraging occurring on the ground than in the vine canopy. This trial was conducted in summer when honeydew excretion by mealybugs was at its peak, thus giving honeydew-feeding ants the opportunity to forage in the vines. The large foraging activity ranges for *L. humile* compared to *C. peringueyi* and *A. custodiens* have implications for bait distribution density. To be economically viable, Daane et al. (2006) recommended that baits be dispensed at 85 baits/ha or less. Our results reveal that at 81 baits/ha (one bait every 9 m), low toxicity baits will reach ±80% of the *L. humile* population, only ±30% of *A. custodiens* workers, and ±20% *C. peringueyi* workers. Lower foraging activities for *A. custodiens* and *C. peringueyi* imply higher bait densities for effective ant control. At high densities, baits of low toxicity will less likely be adopted by growers because of the costs of materials and maintenance involved. However, bait distribution density also depends upon the size of the ant infestation. Thus, all these factors must be considered, including monitoring for pest intensity before determining optimum bait density.

The results further showed that all three species preferred the 25% sugar solution over the other baits offered, except for *A. custodiens* , which preferred tuna. There was a general trend towards preference for liquid or moist baits compared to drier baits, such as dog food and fish meal, for all three ant species. Markin (1970) estimated that 99% of *L. humile* ’s diet consists of honeydew and nectar. Sucrose is the main ingredient of honeydew ( Tennant and Porter 1991 ) and forms a significant part of nectar ( Baker and Baker 1985 ). Baker et al. (1985) carried out food preference trials on *L. humile* and concluded that this ant preferred 25% sugar solution or honey over other solid protein based foods like tuna. Silverman and Roulston (2001) compared the consumption of gel and liquid sucrose formulations by *L. humile* , and results indicated that this species preferred foraging on the gel formulations, which was not supported by our study, although agar and honey were more preferred over drier baits. *Crematogaster peringueyi* is a vineyard ant that forages on mealybug honeydew ( Kriegler and Whitehead 1962 ; Addison and Samways 2000 ), which also explains its attraction to sugar and honey baits during the food preference tests. *Anoplolepis custodiens* is a highly predatory ant species that preys on many insects in South Africa ( Steyn 1954 ; Löhr 1992 ). Its predatory nature would account for this ant being most attracted to tuna.

High attractiveness of 25% sugar solution to *L. humile* and *C. peringueyi* indicates that liquid sugar baits are the most appropriate attractants/carriers for baiting these two species in future pest control. This bait matrix has the advantage that it is cheap and readily available, but it is prone to drying out (due to sugar crystallization) and fungal attack. Furthermore, when formulating toxicants for baiting *A. custodiens* , wet protein attractants, like tuna, are the most ideal bait matrix for maximum effectiveness and should be taken into account. The number of ant foragers at bait stations significantly increased with time, as would be expected due to pheromone calling and establishment of foraging trails ( Goss et al. 1990 ; Greenberg and Klotz 2000 ).

The results of the present study on foraging activity of different ant species are not definitive. More research needs to be done before we can fully understand bait distribution patterns and bait station density. Foraging behavior of ants depends upon a number of environmental factors, one of which is the availability of food sources. Foraging activity field trials were done in summer when honeydew excreted by *P. ficus* and grape juice from the ripening grape clusters were at their peaks. Therefore, this is the time when ant baits would be at their least effective. We presume that ants forage for shorter distances during this time of the year, since food resources will be highly abundant and in close proximity. Nelson and Daane (2007) hypothesized that ant foraging on 25% sugar water peaks in spring. Rust et al. (2000) showed that *L. humile* foraged 26–60% and 16–40% of available protein in summer and winter respectively, suggesting considerable seasonal differences in activity and bait preferences. Thus, further research should focus on repeat-ing the study in spring and autumn to deduce whether increased labeled bait acceptance will translate to increased bait movement in the vineyard. Furthermore, little is known about *C. peringueyi* intra-and interspecific competition, and thus more research should focus on assessing its dominance or lack thereof, which is one of the factors that will determine this ant’s foraging behavior. Food preference may also differ depending on season, and consequently more research still needs to be done on species’ seasonal food requirements (protein, lipids, and carbohydrates).
